# Formin DAAM1 Organizes Actin Filaments in the Cytoplasmic Nodal Actin Network

**DOI:** 10.1371/journal.pone.0163915

**Published:** 2016-10-19

**Authors:** Weiwei Luo, Zi Zhao Lieu, Ed Manser, Alexander D. Bershadsky, Michael P. Sheetz

**Affiliations:** 1 Mechanobiology Institute, National University of Singapore, Singapore, 117411, Singapore; 2 sGSK Group, Institute of Molecular and Cell Biology, Agency for Science Technology and Research, Proteos Building, 61 Biopolis Drive, Singapore, 138673, Singapore; 3 Department of Biological Sciences, Columbia University, New York, New York, 10027, United States of America; 4 Department of Molecular Cell Biology, Weizmann Institute of Science, Rehovot, 76100, Israel; Karolinska Institutet, SWEDEN

## Abstract

A nodal cytoplasmic actin network underlies actin cytoplasm cohesion in the absence of stress fibers. We previously described such a network that forms upon Latrunculin A (LatA) treatment, in which formin DAAM1 was localized at these nodes. Knock down of DAAM1 reduced the mobility of actin nodes but the nodes remained. Here we have investigated DAAM1 containing nodes after LatA washout. DAAM1 was found to be distributed between the cytoplasm and the plasma membrane. The membrane binding likely occurs through an interaction with lipid rafts, but is not required for F-actin assembly. Interesting the forced interaction of DAAM1 with plasma membrane through a rapamycin-dependent linkage, enhanced F-actin assembly at the cell membrane (compared to the cytoplasm) after the LatA washout. However, immediately after addition of both rapamycin and LatA, the cytoplasmic actin nodes formed transiently, before DAAM1 moved to the membrane. This was consistent with the idea that DAAM1 was initially anchored to cytoplasmic actin nodes. Further, photoactivatable tracking of DAAM1 showed DAAM1 was immobilized at these actin nodes. Thus, we suggest that DAAM1 organizes actin filaments into a nodal complex, and such nodal complexes seed actin network recovery after actin depolymerization.

## Introduction

Actin filaments are one of the major building units of the cytoskeleton, and fill the cell with specialized bundles and networks [1]. In the cytoplasm, the network of actin filaments requires bundling, crosslinking and contracting units to make it cohesive. At a functional level, an intact cytoplasmic actin network is required for motility, cell division and many other cellular processes [2–5]. In addition, the cohesiveness of the cytoplasmic actin network allows the cell to resist cytoskeleton perturbations [6, 7]. Our recent study suggested a nodal organization of the cytoplasmic actin network [8]. The network was formed by actin nodes containing the formin, disheveled-associated activator of morphogenesis 1 (DAAM1), and the crosslinker filamin A (FlnA) that were linked by myosin II filaments.

Formin proteins play critical roles in organizing cellular actin. They nucleate and remain associated with the barbed ends of actin filaments during actin filament elongation (reviewed by Chesarone, 2010 [9]). The formin DAAM1 has been shown to aid in the development of the cardiac [10, 11] and neuronal systems [12–17]. DAAM1 could organize actin by nucleating, elongating [18] and possibly, bundling actin [19]. Compared to the formins mDia1 and mDia2, the DAAM1 FH2 domain shows weaker actin assembly activity [18]. However, the mechanism of DAAM1 regulation of the actin network is not well studied.

DAAM1 has been shown to localize to actin structures such as myosin IIB-containing stress fibers [20] and filopodia shafts [19]. Several studies showed that DAAM1 associated with membrane structures using an imaging method or membrane fractionation: plasma membrane [21, 22], endocytic vesicles [13], synaptic membrane [17], protruding membrane of cortical neurons [15]. The membrane localization of DAAM1 could be a result of plasma membrane recruitment by Wnt-Frizzled-Dishevelled signaling pathway [21], or via its membrane binding domains [23]. The localization of DAAM1 indicates that it has a role in organizing actin structures in cytoplasm, filopodia and membrane, but there is a lack of direct observation of DAAM1’s activity in actin assembly.

In our previous study, DAAM1 was found at the nodes of the cytoplasmic actin network [8]. The inhibition or knock down of DAAM1 did not prevent the formation of the actin nodes but largely reduced the dynamics of the actin nodes. Knock down of DAAM1 reduced the fusion and the fission events of the actin nodes that appeared after mild inhibition of actin polymerization by low concentrations of Latrunculin A. To better understand the role of DAAM1 in organizing actin, we examined whether it was responsible for actin polymerization at nodes and at membrane surfaces.

In this study, we show that actin filaments grow from the DAAM1-containing actin nodes to form asters and further organize into a nodal network. When DAAM1 was cytoplasmic but constitutively active, the cytoplasm was organized by an aster mesh network instead of bundled filamentous network. When DAAM1 was translocated to the cytoplasmic membrane using a rapamycin induction system, actin assembly was significantly higher at the cell membrane relative to the cytoplasm. DAAM1 has a significant immobile fraction in the cell as shown in FRAP experiments; and photoactivatable tracking of PATagRFP-DAAM1. The immobile DAAM1 was concentrated at the actin nodes while diffusive DAAM1 was found in the regions between the actin nodes. With these results, we hypothesize that DAAM1 organizes short actin filaments into a complex. These complexes are the seeds for actin network recovery upon perturbation.

## Results

### The N- and C-terminus of DAAM1 play roles in membrane localization

**Fig 1:**
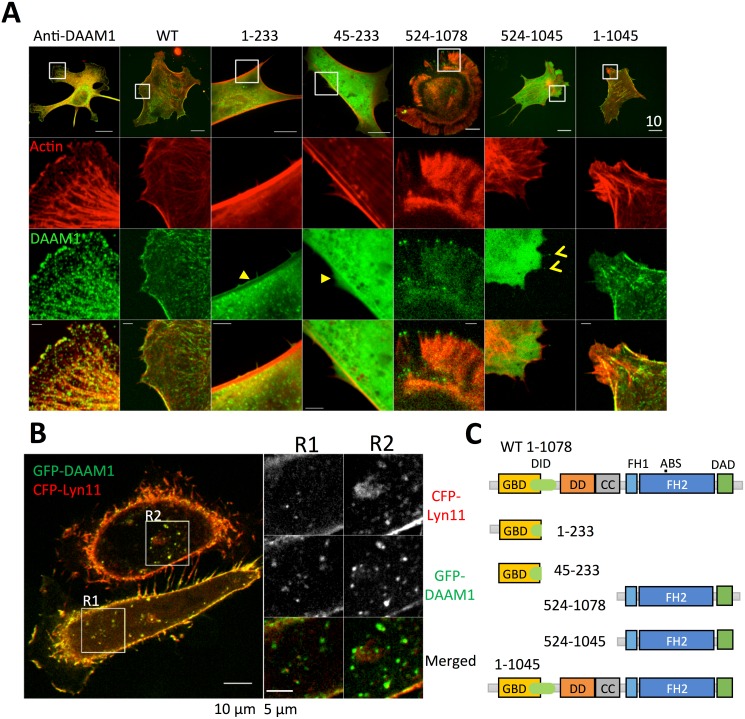
DAAM1 localizes to both membrane and actin cytoskeleton. (A) Localization of endogenous DAAM1 transfected GFP-DAAM1 and the mutants. DAAM1 localized to both cytoplasm and cell edge. Comparing N-terminus mutants, construct lacking the first 45aa did not show membrane localization (yellow triangle). Comparing C-terminus mutants, construct lacking the last 33aa showed less filopodia and filopodia tip localizations (yellow ^). (B) Middle section of live-cell confocal image showed the colocalization of bright DAAM1 puncta with membrane structure. HeLa cells were transfected with GFP-DAAM1 WT and CFP-Lyn11. Two regions of the image are enlarged. (C) Domain structures of DAAM1 and its mutants.

DAAM1 localizes to a subset of stress fibers, filopodia shafts, endocytic vesicles and plasma membranes in cultured cells [13, 15, 17, 19–21]. In mouse embryonic fibroblasts (MEF) and HeLa cells, DAAM1 patches colocalized with actin structures in both cytoplasm, cell membrane and intracellular vesicles, as shown by immunofluorescent labeling or transfection of GFP wild type DAAM1 (Fig 1A and 1B).

Both the N- and C-terminal regions of Diaphanous-related formins are implicated in their localization [23, 24]. Previous work has shown that residues 1–440 of DAAM1 was responsible for its ability to associate with the actomyosin network [20]. A smaller DAAM1 (1–233) construct was clearly localized to the both the cytoplasm and plasma membrane (arrowhead in Fig 1A, column 3). By contrast, DAAM1 (45–233) did not associate with the plasma membrane (arrowhead in Fig 1A, column 4), suggesting that residues 1–45 contain a membrane-localizing signal. Visual inspection indicated two cysteines 16/17, which are conserved in vertebrate DAAMs, are highly likely to be palmitoylated, and would represent a reversible means of DAAM1 membrane localization.

Basic clusters within the last 40 residues at the C-termini of diaphanous-related formins have been invoked as PIP2 interaction sites at the plasma membrane [23]. DAAM1 and DAAM2 contain a conserved C-terminal region not found in other formins that includes polybasic cluster KLKRNRKR. The DAAM1 (524–1078) construct that contains FH1/FH2 and this basic region is constitutive active (ie lacking autoinhibitory interaction between DID and DAD domains). Expression of DAAM1 (524–1078) generated both filopodia and lamedilpodia and was found localized to the tips of filopodia (Fig 1A, column 5). However, the DAAM1 (524–1045) that lacks the last 33 residues and basic cluster was inactive in this respect, remained cytoplasmic (Fig 1A, column 6). Occasional observation of filopodia tip localization of DAAM1 (524–1045) was likely due to the interaction between FH2 domain and actin filaments (Fig 1A, < in column 6). This data is consistent with previous observation that DAAM1 residues 1056–1059 function in tandem with the FH2 domain to promote actin nucleation [25]. However the auto-inhibited DAAM1 (1–1045) mutant lacking the last 33 residues localized to the actomyosin network similarly to the full-length DAAM1 [26].

The above results suggested that wild type DAAM1 localizes to the plasma membrane and is enriched in regions on the ventral plasma membrane with actomyosin stress fibers. Residues 1045–1078 contribute to the membrane localization of constructs that include the C-terminal half of DAAM1 containing the catalytic FH2 domain.

### Actin filaments growth from DAAM1-containing actin nodes in the cytoplasm and the plasma membrane

**Fig 2:**
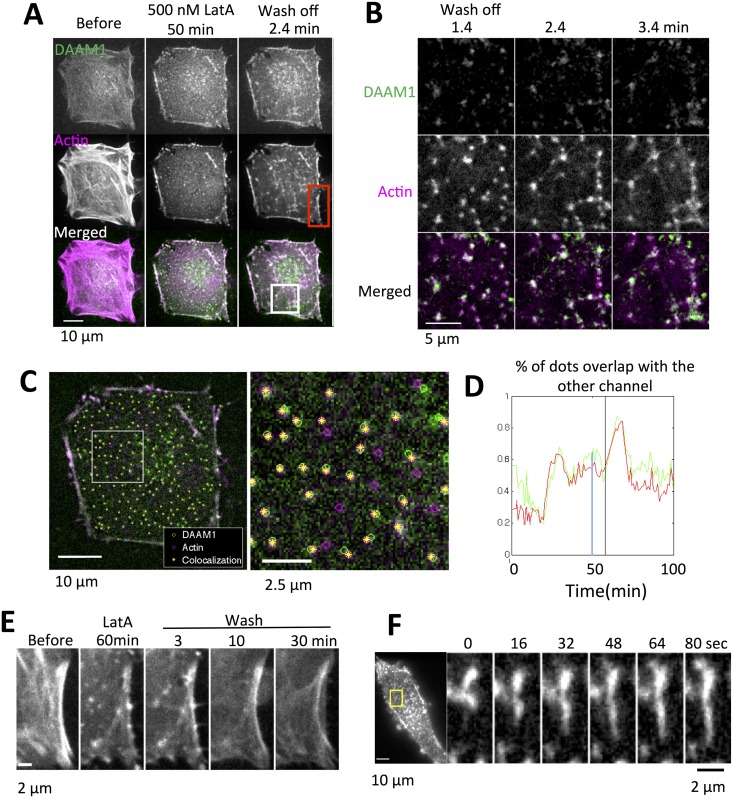
DAAM1 assembled actin in cytoplasm and membrane upon LatA wash off. (A) Actin filament grew from the DAAM1-containing nodes upon LatA wash off. HeLa JW cells were transfected with Ruby-Lifeact and GFP-DAAM1 24 hours before imaging. 500 nM LatA was first added to the cells. Wash off was done by replacing the LatA-containing medium to normal DMEM after 3 times washing. (B) Enlarged area from the white box in (A) showed the details of DAAM1 dots and actin nodes localization in the first few minutes after LatA wash off. (C) Imaris tracking of WT DAAM1 dots and actin nodes at 50 minutes of 500 nM LatA treatment showed 55% of DAAM1 dots was colocalized with actin nodes, and 50% of actin nodes were colocalized with DAAM1 dots. Green: DAAM1 dots. Magenta: actin nodes. Yellow: co-localization of DAAM1 and actin. (D) The percentage of DAAM1 dots associated with actin nodes (green line) and actin nodes to DAAM1 (red line) changed over time. The blue line indicates the quantification time shown in (C). The black line indicates LatA washed off time. (E) Enlarged area from the red box in (A) showed the cell edge actin deformation during the course of LatA treatment and wash off. (F) Actin filament grew from the nodes with the speed of 25.2 ± 12.4 nm/sec.

Treatment of MEF or HeLa JW cells with LatA at concentrations ranging from 200 nM to 800 nM resulted in a biphasic alteration of the actin pattern as we previously reported: in phase 1, retraction and the subsequent disappearance of actin stress fibers was observed; whereas in phase 2, numerous micron-sized actin nodes appeared (Fig 2A).

The LatA effect was reversible (S1 Movie): upon washing off LatA with cell culture medium, actin filaments grew from the DAAM1-containing actin nodes (Fig 2B) within 2 to 3 minutes, producing an aster-shaped network. Not all the actin nodes and DAAM1 dots colocalized. Using Imaris spot tracking, the percentages of GFP-DAAM1 dots that overlapped with actin nodes was 50–80% (Fig 2C and 2D).

Actin recovery after LatA treatment can also be observed at the cell membrane. LatA treatment resulted in beads-on-a-string actin structures, resembling a 1-dimensional network of asters. LatA wash off resulted a slight intensity increase at the edge of the cell: the edge intensity measured at 60 minutes after LatA wash off among 6 cells was 1.07 ± 0.17 times higher compared to the intensity at the end of LatA treatment, which was far from the intensity before LatA treatment. The shape of the cell edge could also be considered as another measure of actin recovery after LatA washout. The edge changed from concave before LatA treatment, to straight at the end of LatA treatment, and back to concave shape after wash off (Fig 2E). This observation using fibronectin coated coverslip differed from another study using a patterned substrate [27], in which cell edges became more concave after treatment with Y27632. In the example shown in S1 Movie, the straightening of the actin edge appeared when the attached actin stress fibers detached. Upon LatA washout, the deformation of the actin edge occurred during the actin aster network formation and was coupled with lamellipodia protrusion and retraction. This was taken as a sign of fluctuating membrane tension and active actin polymerization[28]. Both actin filament staining intensity measurements and shape change at the cell edge indicated that the actin recovered at the cell edge, after LatA wash off.

The F-actin filament growth rate was estimated from fast time lapse analysis of RFP-Lifeact after LatA wash off, before the filaments merged together. The estimation based on the gradient derived from kymographs gave an elongation rate of 25.2 ± 12.4 nm/sec (in 4 cells, 14 randomly chosen filaments), equivalent to 9 subunits/sec (Fig 2F).

These results showed that wild type DAAM1 plays a role in actin assembly after LatA wash off. The actin filaments grew from the DAAM1-containing nodes in the cytoplasm, as well longer structures at the plasma membrane.

### An active, cytoplasmic form of DAAM1 caused nodal actin filament arrays

**Fig 3:**
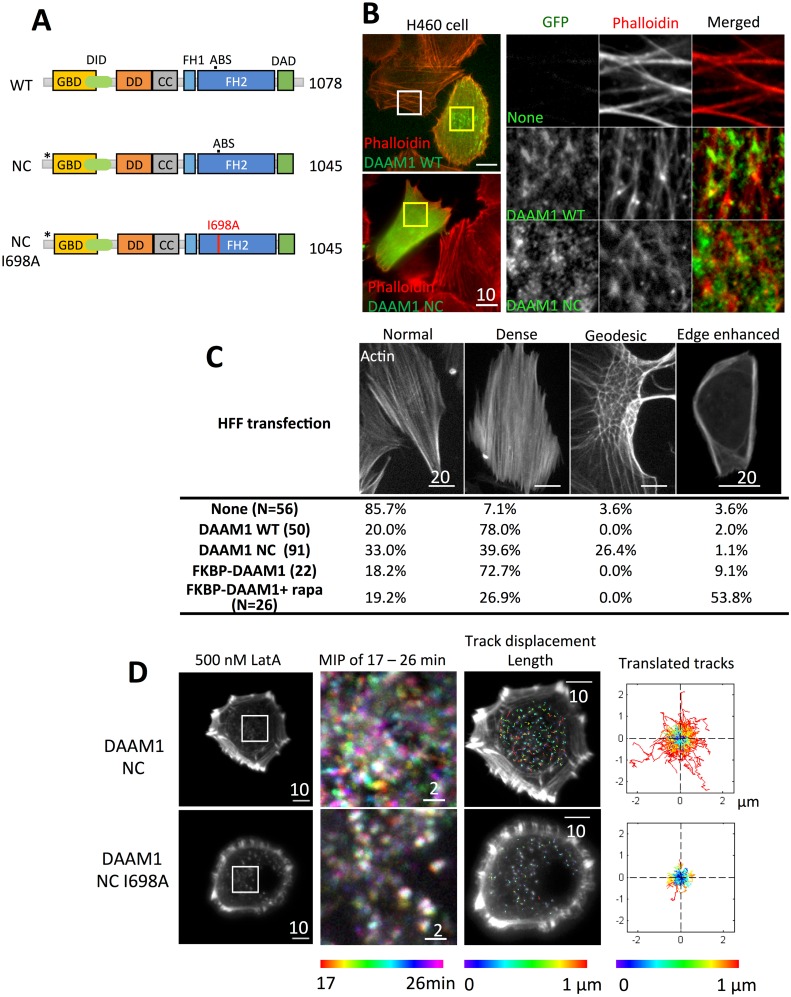
Actin structures change upon DAAM1 wild type (WT) and NC mutant transfection. (A) Schematic illustrations of the two constructs. *: C16-17A mutation. (B) H460 cells were transfected with either DAAM1 WT or NC mutant 24 hours before fixation then stained with phalloidin. Epifluorescence images were taken. White box showed the actin structure of untransfected cells, and yellow boxes showed the transfection of respective GFP constructs. Rolling ball background subtraction was performed to the insert to increase the contrast (C) The transfected HFF cells were categorized into 4 groups according to the actin structure. (D) Actin nodes movement in NC or NC I698A transfected H460 cells were compared. The inserts showed temporal-color coded 9-minute maximum intensity projection (MIP), from 17 to 26 minutes after 500 nM LatA treatment. Actin nodes movement was tracked using Imaris. All the tracks were then translated to initial position (x = 0, y = 0) with color-coded track displacement length.

The observation of DAAM1 colocalization with actin in both the cytoplasm and at the cell membrane during actin recovery indicated that it functions in both locations. Therefore, we hypothesized that a cytoplasmic form of DAAM1 would create denser actin network in the cytoplasm, while a membrane form of DAAM1 would create stronger actin networks at the cell membrane. To create a mutant of DAAM1 that lacked the membrane localization domains, both N and C-terminal regions were altered. This DAAM1-NC mutant had C16-17A mutations at the N-terminus to remove the palmitoylation sites, and the poly-basic cluster KLKRNRKR was deleted in the C-terminal region to decrease the interaction with PIP2 (Fig 3A).

The DAAM1-NC mutant created actin meshwork with short filaments in the cell lines tested in this study, including MEFs, human foreskin fibroblast (HFFs), HeLa JW cell line, and the H460 non-small cell lung cancer cell line that is deficient of DAAM1 [20]. Compared to the well-organized actin structure in DAAM1 (WT)-transfected cells, DAAM1-NC-transfected cells appeared to have shorter filaments and a mesh type of actin structure (Fig 3B and 3C, S1A and S1B Fig). In H460 cells, the WT-transfected cells were similar but with greater actin filament density compared to the untransfected cells (Fig 3B). On the contrary, almost all the DAAM1-NC-transfected cells showed a mesh type of actin network (Fig 3B). Interesting polygonal actin arrangements surrounding the nucleus region appeared in DAAM1-NC transfected fibroblasts. Such actin structure, called geodesic actin structure, was first described in 1979 [29] and often observed in human trabecular meshwork cells [30–32]. In MEFs, 3 out of 36 cells with GFP-DAAM1-NC transfection showed the geodesic actin structure while none in the 80 DAAM1-WT transfected cells. In HFFs, 78% of WT-transfected cells showed dense actin filaments but no geodesic actin. On the other hand, the geodesic actin structure was present in a significant fraction (26.4%) of GFP-DAAM1-NC containing HFFs (Fig 3C).

After LatA treatment, DAAM1-NC mutant localized to the center of the actin nodes. Thin actin filaments extended from the actin nodes, forming aster structures in MEFs (S1C Fig). Since DAAM1 siRNA did not completely remove endogenous DAAM1[8], H460 cells that naturally lacked DAAM1[20] were used to test the actin assembly activity of DAAM1-NC mutant. H460 cells showed actin nodes when treated with mild LatA, but few were actively moving. However, H460 cells with DAAM1-NC transfection formed active moving actin nodes upon LatA addition (Fig 3D, S2 Movie). The actin nodes were often connected by short actin filaments. This indicated that DAAM1-NC mutant was capable of assembling actin filaments like the wild type. Mutation of isoleucine^698^ to alanine (I698A) was shown to abolish the actin assembly activity of Daam1 [18]. DAAM1-NC mutant was further modified to I698A. DAAM1-NC (I698A) transfected H460 cells exhibited static actin nodes after LatA treatment, similar to the untransfected H460 cells. Quantification was performed to compare the actin node localization in 9-minute intervals during LatA treatment. When the actin nodes were actively moving, the maximum intensity projection (MIP) with temporal color code showed a spectrum of colored nodes in DAAM1-NC transfected cells. On the other hand, DAAM1 NCI698A transfected cells contained immobile actin nodes, resulting in white nodes in the MIP image. Imaris tracking of 33 frames, 9 minutes of the S2 Movie showed that the actin nodes showed more mobility in DAAM1-NC transfected cells than in DAAM1-NC (I698A) cells (Fig 3D).

These results indicated that the DAAM1-NC (a cytoplasmic form of DAAM1), does exhibit actin assembly activity. In addition, it enhanced the nodal actin organization by polymerizing short actin filaments in the cytoplasm.

### Actin depolymerization is required for DAAM1 translocation from actin nodes to the cell membrane

Under normal condition, wild type DAAM1 associates with various actin structures in both cell membrane and cytoplasm. Since the cytoplasmic form of DAAM1 resulted actin meshwork in the cytoplasm, we further speculate that a membrane form of DAAM1 could create dense actin network at the cell membrane.

**Fig 4:**
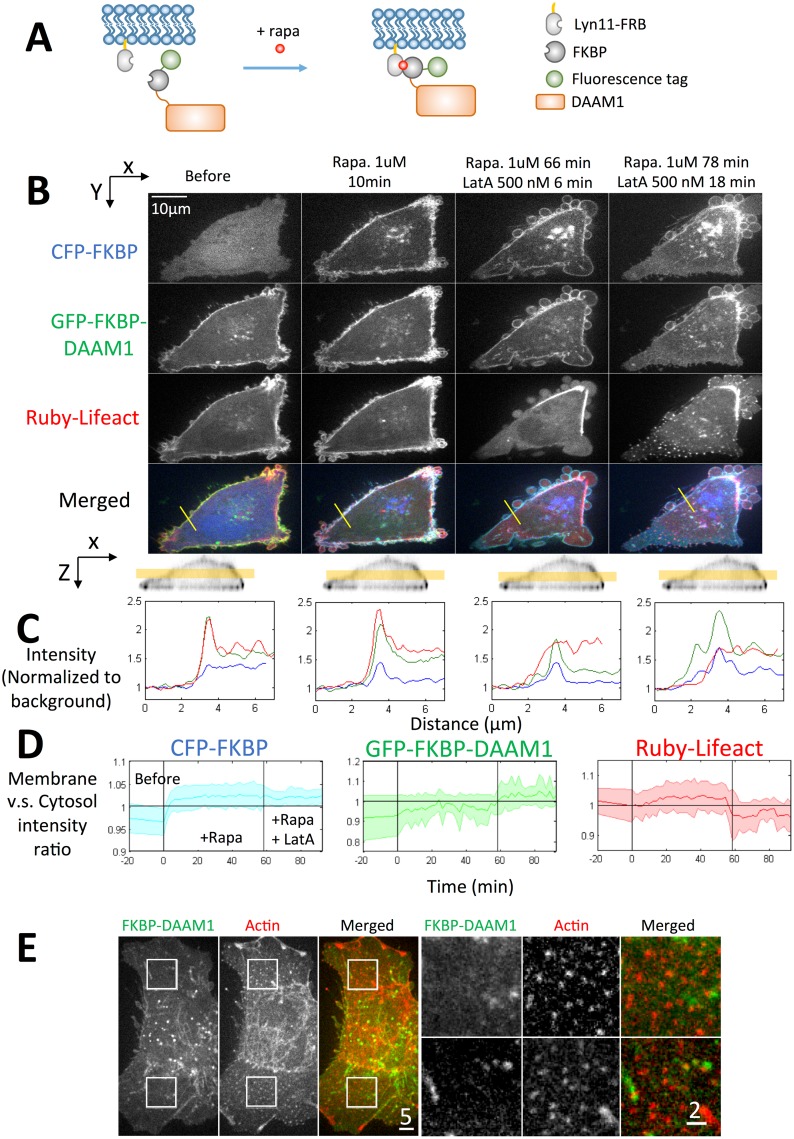
Rapamycin-trigged membrane translocation of DAAM1 requires actin depolymerization. (A) Schematic diagram of inducible translocation of DAAM1. (B) Rapamycin addition induced fast translocation of CFP-FKBP but minor translocation of GFP-FKBP-DAAM1. HeLa cell was transfected with Lyn11-FRB, CFP-FKBP, GFP-FKBP-DAAM1, Ruby-Lifeact and treated with 1 uM Rapamycin and 500 nM LatA as indicated. The middle confocal section of the cell was shown as indicated by the yellow bar at the bottom. (C) Intensity of the yellow line in the merged image in (B) is normalized to the background. Blue: CFP-FKBP. Green- GFP-FKBP-DAAM1. Red: Ruby-Lifeact. (D) Quantification of cell membrane vs. cytosol intensity change. The membrane area of the cell was defined as a 1 μm-thick area from the cell outer surface of the confocal z-stack. The intensity quantification was done by averaging 7 cells. The bold line is the mean value and the shade represents the standard deviation. (E) Combination of 1μM rapamycin and 500nM LatA treatment for 1 hour eliminated the localization of GFP-FKBP-DAAM1 at actin nodes.

A strategy of inducible membrane translocation of DAAM1 was used. Rapamycin triggered the heterodimerization between a rapamycin-binding domain of mTOR (FRB) and the FK506-binding protein (FKBP) [33]. The FRB was plasma membrane targeted using a Lyn N-terminal sequence (termed Lyn11-FRB), while the FKBP was linked to DAAM1. Addition of rapamycin to the culture media then triggered the binding between FKBP and FRB, causing DAAM1 to bind to the plasma membrane. This process was illustrated in a schematic diagram (Fig 4A) and was referred as “knock sideways” in comparison with knock down approach[34].

Unexpectedly, rapid DAAM1 translocation to membrane after rapamycin treatment did not occur. With 1 μM rapamycin treatment, CFP-FKBP, which served as an internal positive control of FKBP-FRB binding, concentrated at the membrane within 4 minutes (Fig 4B). In contrast, GFP-FKBP-DAAM1 only showed a minor intensity increase at the membrane region even with 1-hour rapamycin treatment (Fig 4B, 4C and 4D). With extended incubation (>20 hours) of rapamycin, FKBP-DAAM1 showed increased membrane localization. However, the toxic effect of rapamycin was clear with long-term incubation.

**Fig 5:**
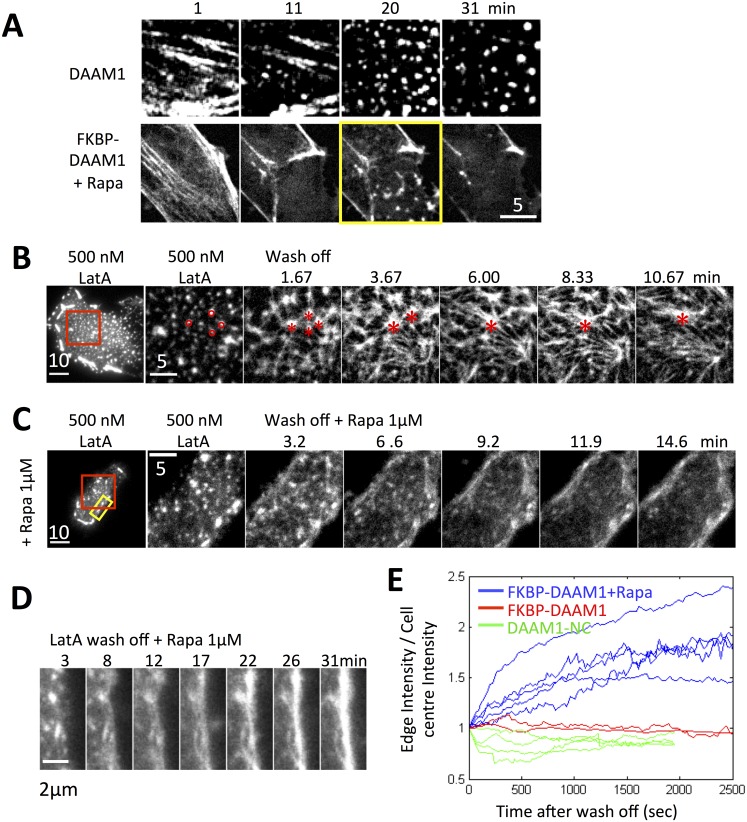
Membrane translocation of DAAM1 resulted in transient actin nodes upon LatA treatment and peripheral actin filament assembly upon LatA wash-off. (A) HeLa JW cells time lapse images of the actin node dynamics in control (transfected with WT DAAM1 and Lifeact) and inducible translocation system (transfected with GFP-FKBP-DAAM1, Lyn11-FRB and Lifeact). Actin nodes appeared transiently 20 minutes after LatA treatment in the cells with DAAM1 translocate to the membrane (highlighted in yellow box). The image corresponds to S3 Movie. (B) The cell was treated with 500 nM LatA then wash off using culture medium. The red box region is enlarged on the right. Four actin nodes in the last frame of LatA treatment were marked in red circles. After wash-off LatA, actin polymerization occurs. These nodes became asters and marked as “*” in red. Images were background subtracted to increase the contrast. (C) The cell was treated with a combination of 1 μM rapamycin and 500 nM LatA then wash off LatA using 1 μM rapamycin-containing medium. The red box region is enlarged on the right. The HeLa JW cells were transfected with GFP-FKBP-DAAM1, Lyn11-FRB and Lifeact in (B) and (C). (D) The cell edge area is enlarged from the yellow rectangular in (C) to show the actin intensity change over time after LatA wash off. (E) The intensity ratio between membrane and cytoplasmic region after LatA wash off. The membrane area was defined as 1 μm-thick area from the cell outer surface.

Actin nodes occurred after co-treatment of LatA and rapamycin. However, DAAM1 did not localize with these actin nodes (Fig 4B and 4E). An interesting finding was that the actin nodes were transient in the rapamycin-LatA treatment system. The actin nodes appeared 15 to 20 minutes after LatA treatment and last for 4 to 7 minutes (observation of 5 cells, Fig 5A and S3 Movie). In contrast, active moving actin nodes in non-rapamycin system lasted much longer before cell underwent apoptosis, even for hours [8].

Thus we suggest that LatA caused dynamics of the actin nodes and the release of DAAM1 to the soluble pool. Normally the soluble DAAM1 recycled back to the actin nodes. With rapamycin induced membrane translocation, the soluble DAAM1 was translocated to the cell membrane. Actin nodes that lack DAAM1 cannot last long and disappeared soon after they formed.

### Membrane translocation of DAAM1 caused actin assembly at membrane

Quantification of membrane intensity change indicated that GFP-FKBP-DAAM1 translocated to the membrane under co-treatment of rapamycin and LatA (Fig 4D). We further tested the actin assembly activity of DAAM1 by washout of LatA.

Cells transfected with Lyn11-FRB, GFP-FKBP-DAAM1 and Ruby-Lifeact were used to study actin network recovery after LatA washout in either the presence or absence of rapamycin (Fig 5B–5E). The LatA treatment was stopped after actin nodes formed and GFP-FKBP-DAAM1 moved to the membrane, typically 15 to 20 minutes after LatA addition. Initially, the actin polymerization after LatA washout was primarily at the membrane where short cytoplasmic actin filaments formed rapidly (Fig 5C, 3.2min). However, these filaments were not able to grow into a network. The filamentous actin intensity at the cell center went down, while the membrane intensity went up (Fig 5D and 5E). Quantification established the reproducibility of this actin polymerization pattern for cells in rapamycin medium (Fig 5E, blue curves). Thus, the mobilization of DAAM1 from nodes by LatA enabled GFP-FKBP-DAAM1 to be sequestered away from the cytoplasmic nodes, and that resulted in the absence of nodal actin growth in cytoplasm during the LatA washout.

In contrast, washout of 500 nM LatA caused actin filament assembly from the actin nodes in the absence of rapamycin, forming aster like structures (Fig 5B, 1.67 min). The filaments from neighboring actin nodes then connected to each other to form a network. Some asters merged and the network increased in density over time (Fig 5B, 3.67–6 min). The actin node centers disappeared as the actin filaments grew longer (Fig 5B 13 min.). The network transited from aster/orthogonal organization into parallel/anti-parallel filament bundles. At the cell membrane, the actin bundles did not show a significant increase in intensity on average (Fig 5E, red curves). Further, in the cells transfected with the DAAM1 NC mutant, which was the cytoplasmic form of DAAM1, actin filament recovery at the cell membrane was less (Fig 5E, green curves).

Together, the results indicated that DAAM1 localization to the nodes was required to sustain the actin nodes after LatA washout. Once bound to cell membrane, DAAM1 only induced actin assembly at the cell membrane after LatA washout.

### Fluorescence recovery after photobleaching (FRAP) showed there is a significant portion of immobile DAAM1

**Fig 6:**
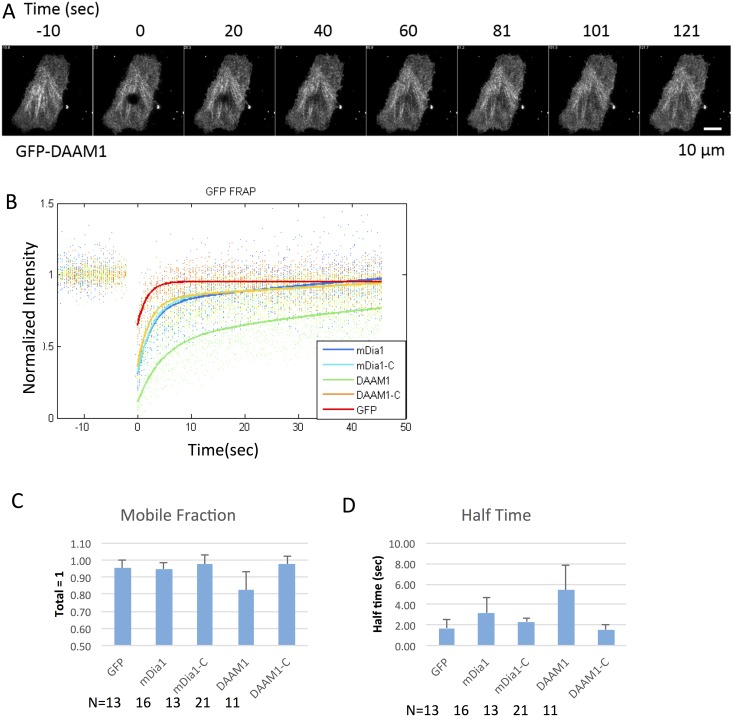
Fluorescence recovery after photobleaching (FRAP) showed there is a significant portion of immobile DAAM1. (A) FRAP images of GFP-DAAM1. Scale bar: 10 μm. (B) The recovery curves of GFP vector, GFP-mDia1, GFP-mDia1C (543–1192 aa), GFP-DAAM1 and GFP-DAAM1C (524–1078 aa). DAAM1 recovered significantly slower than the others. (C) Mobile fraction measurements from all the samples. (D) Half time measurements from all the samples.

To determine if DAAM1 was normally dynamic in the cytoplasm, FRAP was used to measure diffusion rate and bound fraction of DAAM1 molecules in comparison with GFP alone and GFP-mDia1. A circular region of interested (ROI) was chosen as the FRAP region (Fig 6A), and the same bleaching and acquisition settings were used for all samples. A reaction-diffusion model containing two exponential terms was fitted to the fluorescence recovery curves. Mobile fraction and recovery half time were estimated accordingly (Fig 6C and 6D). The results were further tested for the significance of differences between samples using a T-test.

In the case of GFP alone, the known diffusion coefficient was in the range of 25 ~ 30 μm^2^/s in the cytoplasm [35]. For DAAM1, the recovery half time was approximately 3 ~ 8 fold slower than GFP. The effective diffusion coefficient of DAAM1 was then estimated in the range of 3 ~ 10 μm^2^/s and it was also significantly slower than mDia1. The effective diffusion coefficient was later used to estimate the maximum diffusion radius in photoactivation experiments. Further, the immobile fraction of DAAM1 was significantly greater for DAAM1 (~20%) than for GFP or DAAM1-C (< 5%). Thus, DAAM1 was partially immobilized in the cytoplasm as evidenced by the slower diffusion rate and high immobile fraction.

### Photoactivatable tracking of PATagRFP-DAAM1 shows actin dependence of dispersion

High variations in the FRAP results in different regions of single cells indicated that regional binding interactions were occurring. Because of the known interactions of DAAM1 with actin, inhomogeneities in actin filament organization possibly explained the FRAP variations. To test if DAAM1 was immobilized preferentially in actin filament rich regions, we used a combination of photoactivatable tracking of PATagRFP-DAAM1 and FRAP of GFP-β-actin to simultaneously follow DAAM1 molecular movements and actin filament density. PATagRFP-DAAM1 was easily expressed in HeLa cell at a slightly lower level compared to GFP-DAAM1 (S2 Fig).

A typical example of simultaneous FRAP and photoactivation was shown in S3A Fig The PATagRFP-DAAM1 images collected for 1 second were projected in color-coded MIP image to see the dispersion of the fluorescence after photoactivation (S3A Fig). The GFP-β-actin was bleached by the intense 405 nm laser and gradually recovered over 90 seconds, a much longer period of time compared to the duration of visible photoactivated images using stream acquisition (Kymograph in S3A Fig).

**Fig 7:**
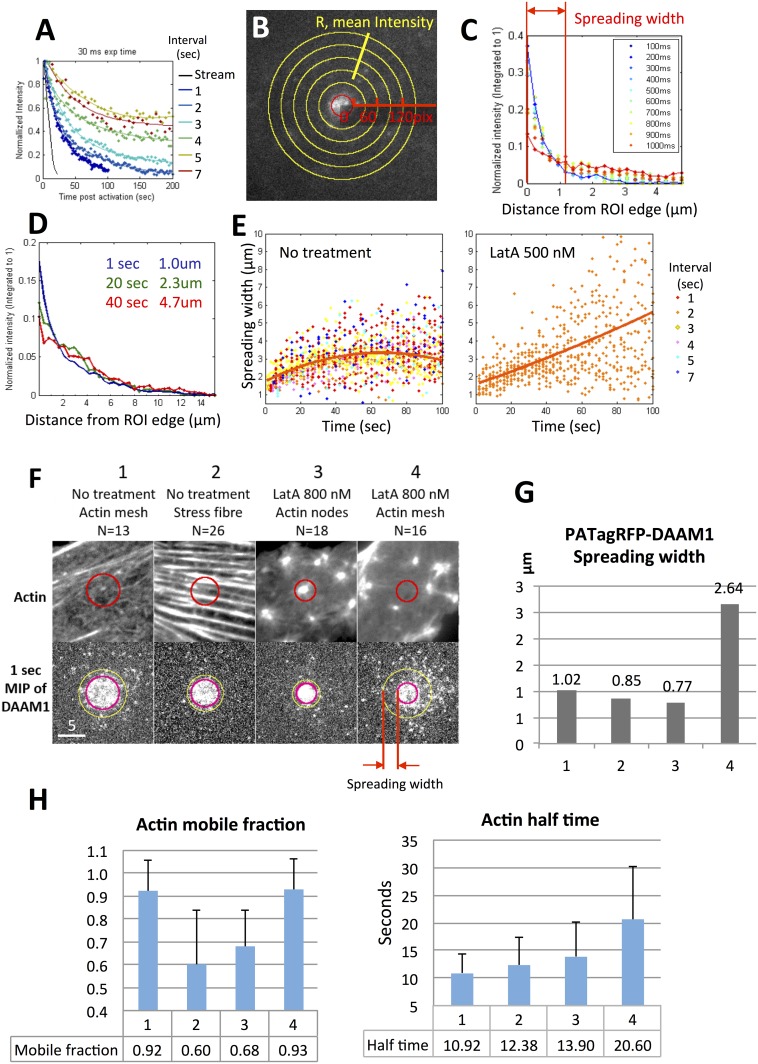
Photoactivatable tracking of PATagRFP-DAAM1 showed the dispersion of DAAM1 molecules was related to actin structures and drug treatments. (A) PATagRFP-DAAM1 intensity dropped exponentially with increased exposure time. With constant 30 ms exposure time, various intervals between frames were used. The curves were exponential fits of the measured intensity except the data from stream acquisition. (B) Demonstration of the intensity over distances measurement of PATagRFP-DAAM1. Given the R as the distance away from the edge of ROI, the mean intensity between (R, R+ΔR) ring regions was measured. (C) A plot of measurement in (B) with multiple time points post-activation. The spreading width is defined as the half-maximum width at time = 1 sec. (D) The spreading width extended further for longer time post-activation. (E) The PATagRFP-DAAM1 spreading width vs. post-activation time in cells with and without LatA treatment. The orange line is the overall trending line for data collected in various time intervals. Note that LatA treatment samples were acquired only with 2 second interval due to the time limits of drug treatment. (F) The FRAP and photoactivation samples were categorized into 4 groups according the actin structures and the drug treatments. DAAM1 molecules dispersion was different in different groups. The first row is the typical GFP-β-actin structure 1 frame before the FRAP/photoactivation. The second row is the 1 sec-maximum intensity projection of all the samples in each group. Scale bar: 5 μm. (G) The spreading width measurement of each group corresponding to the group in (F). It is an estimation using the data points from all the samples. No error bars are available. (H) The actin FRAP measurements of each group corresponding to each group in (F).

Bleaching and diffusion were two reasons for intensity decay of PATagRFP-DAAM1. In order to track the fast molecular dynamics, streaming acquisition was used. However, severe bleaching occurred in streaming acquisition (Fig 7A, S3B Fig). The decay rate reduced as the interval increased. Bleaching was negligible only with a long interval (5 to 7 seconds) between each 30ms acquisition. Therefore, we compared measurements with fixed acquisition parameters to determine the diffusion of PATagRFP-DAAM1.

The movements of the PATagRFP-DAAM1 molecules were captured using 25Hz frame rate and were tracked using Imaris software (S3C and S3D Fig). The average speed of the PATagRFP-DAAM1 spots was 5.17 μm/s with a moderate searching radius of 1 μm. More than 90% of the tracks did not move more than 2 μm but some spots appeared at regions 5 μm away from the activation region as soon as 80 ~ 240 ms post activation (S3C Fig, right most figure, highlighted in red circles). These spots co-localized on stress fibers with 1 ~ 2 μm spacing away from each other, consistent with super-resolution images[8]. Some spots were observed to last up to 5 seconds without moving (S3E Fig). Among the long-lasting spots (>1 second post-activation), 33% to 50% were colocalized with actin nodes that appeared after LatA treatment.

Due to the short lifetime and possible blinking of the fluorescence tag, the tracking algorithm from Imaris became unreliable for the short tracks or with long intervals between frames. An alternative method was developed to quantify the photoactivation images using Matlab as described in Materials and Methods (Fig 7B and 7C). A “spreading width” (Fig 7C, red arrow) was a measurement of how far the DAAM1 molecules spread after a certain interval post-activation. With longer intervals between each acquisition, the spreading width estimation can be extended for longer times post-activation. Different acquisition intervals showed a similar trend of spreading width in untreated cells. Interestingly, DAAM1 did not spread more than 7 μm post-activation in cells without LatA treatment, but spread much further in cells treated with 500μm LatA for 15 to 20 minutes (Fig 7E).

The spreading width reflected the dispersion rate of DAAM1 molecules either through directed movement of DAAM1 molecules, and/or the diffusion of the molecules. Samples were grouped according to their actin structure and the drug treatment. There were in total 4 groups (Fig 7F): (1) actin mesh in the untreated samples, (2) stress fibers in the untreated samples, (3) actin nodes in the LatA-treated samples and (4) actin mesh between the nodes in the LatA-treated samples. Three measurements, including DAAM1 spreading width at 1 second post activation, GFP-β-actin FRAP mobile fraction and FRAP recovery half time, were listed in Fig 7G and 7H. In cells without any drug treatment, the spreading width in regions with strong stress fibers was slightly smaller (group 2, 0.85 μm) than the regions without clear stress fibers (group 1, 1.02 μm). In LatA treated samples, the DAAM1 molecules were highly condensed at actin nodes, with small spreading width (group 3, 0.77 μm). On the other hand, in regions between the actin nodes in LatA treated cells, DAAM1 molecules appeared to be diffusive, spreading 2.64 μm away from the edge of ROI in 1 sec (group 4).

Among the four groups of samples, certain pairs of parameters had high correlations. The correlation coefficient between GFP-β-actin mobile fraction and GFP mean intensity was negative, -0.889. Most of the high GFP intensity samples showed dense actin structures, either stress fibers or dense actin nodes. This suggested that these dense actin structures contain less mobile GFP-β-actin. The correlation coefficient between GFP-β-actin mobile fraction and the normalized PATagRFP-DAAM1 intensity in the first frame post-activation within the ROI region, was -0.801. This indicated that at the regions where more PATagRFP-DAAM1 was found, there was less mobile GFP-β-actin. The correlation coefficient between GFP-β-actin mobile fraction and PATagRFP-DAAM1 spreading width was 0.620. This suggested when there was more mobile actin, the PATagRFP-DAAM1 spread further. The correlation between recovery half time and spreading width was 0.611, which suggested slower actin recovery time was positively related to further spreading of PATagRFP-DAAM1. Last but not least, GFP-β-actin mobile fraction was independent of the recovery half time, as the correlation coefficient was 0.021.

## Discussion

Formin DAAM1 is a key factor in assembly of the cytoplasmic actin nodal network. Our previous study [8] showed that DAAM1 localized at the multi-nodal actin array that appeared after LatA treatment. However, small molecule inhibition or RNAi silencing of DAAM1 did not prevent the appearance of actin nodes but significantly reduced the actin node movement. Using protein sequence analysis and the rapamycin inducible membrane localization system, we generated two forms of formin DAAM1: DAAM1-NC mutant, a cytoplasmic form, and FKBP-DAAM1, the membrane form. Using these mutants and photo-perturbation techniques, we clarify DAAM1’s function in cytoplasmic actin assembly, and show that localization of DAAM1 affects its function.

The cytoplasmic form of DAAM1 causes actin assembly in the cytoplasm. After modifications at both N- and C-terminus, DAAM1-NC mutant localized at the actin nodes after LatA treatment similar to the wild type (S1C Fig). When DAAM1-NC mutant is transfected into H460 cell, a cell line known lack of DAAM1, DAAM1-NC restores the actin node dynamics after LatA treatment. In contrast, after an additional mutation in the FH2 domain I698A, DAAM1-NC (I698A) cannot restore the actin node movement in H460 cells (Fig 3D and S2 Movie). Thus DAAM1-NC mutant is active as it is capable of actin polymerization. Overexpression of this mutant results in dense, aster shapes of the cytoplasmic actin network in H460 cells (Fig 3B), and increases in geodesic actin networks in HFF cells (Fig 3C, S1A and S1B Fig), while overexpression of wild type DAAM1 only increases the density of actin filaments. Therefore, we conclude that removal of membrane localization domains of DAAM1 results in strong cytoplasmic aster actin network assembly.

On the other hand, the membrane form of DAAM1 weakens cytoplasmic actin network but strengthens membrane actin. The most direct evidence comes from the actin recovery after LatA wash off. Actin assembly in cells transfected with wild type DAAM1 appeared in both cytoplasm and cell membrane, while actin assembly in cells transfected with membrane attached DAAM1 is much stronger at the cell membrane (Fig 5B–5E).

DAAM1 movement to the membrane requires filament disassembly after treatment with LatA. In the rapamycin-induced membrane translocation system, DAAM1 cannot be efficiently translocated to membrane. Depolymerization of actin (via LatA) is required for membrane translocation of DAAM1. These results indicate that DAAM1 may have a strong association with the actin structure. In consistent with these results, the FRAP result shows slower DAAM1 recovery, smaller mobile fraction and large variations in different regions of the cells. The photoactivation results shows that DAAM1 concentrates in the dense actin regions where faster actin polymerization is detected. Bundling of DAAM1 to actin filaments might explain these results. Actin bundling activity of DAAM1 is present in the *Drosophila* isoform dDAAM [36]. Such bundling activity is found in collaboration with fascin, an actin bundling protein [19]. Both studies show that the active form of DAAM1, containing FH1-FH2-C domains, is able to bind to the side of actin filaments.

The rapamycin-induced protein translocation is often termed “knock sideways”[34]. We would like to emphasize the differences between DAAM1 knock down and knock sideways. Actin nodes appeared and last in LatA treated DAAM1 knock down cells, with reduced motility [8], while actin nodes appeared transiently in DAAM1 knock sideways system. Recovery of cytoplasmic actin network was observed in DAAM1 knock down cells after LatA wash off possibly due to the presence of other formins, while recovery of actin was mainly at the cell membrane in the DAAM1 overexpressed, knock sideways system.

We hypothesize that DAAM1 organizes short actin filaments in the cytoplasmic actin nodal network. First, when LatA is present, DAAM1 disassociates from the depolymerized actin filaments. However, we find the localization of DAAM1 at the actin nodes appears after LatA treatment [8]. Under the same conditions, we find drastic differences of DAAM1 molecule movements in the nodal region vs outside the nodal region (Fig 7, group 3 and group 4). Since DAAM1 associates with actin filaments, the actin at the nodal region could be aggregates of many short actin filaments. Furthermore, longer actin filaments are able to grow from such DAAM1-short actin filament complexes (Fig 2B), suggesting that nodes are seeding actin polymerization.

Is the growth of actin filaments at the actin nodes due to the actin polymerization activity of DAAM1 or other formins? We did not have direct evidence. However, the actin assembly at cell membrane upon DAAM1 membrane translocation (Fig 5), and the aster shape or geodesic actin network occurrence upon the transfection of cytoplasmic active form of DAAM1 (Fig 3 and S1 Fig) indicates that DAAM1 catalyzes actin polymerization for the short actin filament assembly.

In conclusion, cells utilize slow actin polymerization machinery with actin bundling activity such as DAAM1 to organize a cytoplasmic nodal actin network. DAAM1 organizes such a network via forming a complex with short actin filaments, instead of utilizing free monomeric actin. The finding in this study further supports our hypothesis that the actin nodes appearing after LatA treatment are not formed *de novo*, but form from the aggregation of pre-existing filaments. The findings in this study also update our previous model [8], that formin DAAM1 is not simply locating at the center of the actin asters to form a complex of its own, but forms a complex with short actin filaments using its actin binding sites outside the FH2 domain. Such organization enables the fast recovery of the cell from actin cytoskeleton disruption within a few minutes, increases the survival of the cell when facing external perturbations such as bacterial toxin or mechanical puncture.

## Materials and Methods

### Plasmids

Truncation and point mutation of DAAM1 plasmids were generated by PCR methods as described before[20]. Expression vectors encoding the following fluorescent fusion proteins were used: EGFP-Lifeact [37] and Lifeact-Ruby (gift from R. Wedlich-Soldner, Max Planck Institute of Biochemistry, Martinsried, Germany), tdTomato–F-tractin (gift from M. J. Schell, Uniformed Services University, Bethesda, Maryland), PATag-RFP (gift from Dr. Vladislav Verkhusha [38]). The PATag-RFP construct containing full length DAAM1 was generated by subcloning DAAM1 from the GFP-DAAM1 plasmid. CFP-FKBP (Addgene #20160), Lyn11-FRB-CFP (Addgene #38003) and Lyn11-targeted FRB (Addgene #20147) were purchased from Addgene. GFP-β-actin was purchased from CLONTECH Laboratories.

### Cell culture and transfection

Immortalized MEFs [39], Human foreskin fibroblasts (HFFs) (ATCC^®^ SCRC-1041^™^, purchased from American Type Culture Collection (ATCC)) and HeLa JW cells [40] were maintained in DMEM high glucose medium (Gibco) supplemented with 10% fetal bovine serum (Gibco), 1% L-glutamine, 1 mM sodium pyruvate and 100IU/mg penicillin-streptomycin (Invitrogen) at 37°C and 5% CO_2_. Human large cell lung cancer cell line H460 (ATCC^®^ HTB-177^™^, purchased from ATCC) were maintained in ATCC-formulated RPMI-1640 Medium (Catalog No. 30–2001).

Cells were transfected with DNA plasmids using electroporation (Neon transfection system, Life Technologies) following the manufacturer’s instructions. The electroporation condition for MEF consists of 1 pulse of 1700 V for 30 ms; for HFF, 2 pulses of 1150 V for 30 ms; for HeLa JW, 2 pulses of 1005 V for 35 ms. H460 cells were transfected using Lipofectamine^®^ 3000 (Thermo Fisher Scientific) following the manufacturer’s instructions.

### Chemicals, antibodies and immunoblotting

All reagents used in this study are chemical grade and were purchased as indicated. Latrunculin A (L5163) and Rapamycin (R8781) were purchased from Sigma, Singapore. The small molecule formin inhibitor, SMIFH2 (Cat. No. 5992446) was purchased from ChemBridge Corporation, San Diego, USA. All chemicals were dissolved in DMSO and then use at the indicated concentrations.

Polyclonal DAAM1 antibody was used in immunofluorescence experiments. The affinity purified rabbit polyclonal antibody against human DAAM1 was raised as described before [20]. Alexa-conjugated secondary antibodies were purchased from Therom Fisher Scientific. DAAM1 antibody used for western blot was purchased from Abcam (ab ab56951). Tubulin antibody for western blot was from Sigma (T5168). GFP antibody was from Santa Cruz (sc8334). Wesetrn blot analysis was carried out as indicated in[8].

### Immunofluorescence

Cells are fixed and simultaneously permeabilized at 37°C in a mixture of 3% paraformaldehyde, 0.2% glutaraldehyde and 0.25% Triton-X100 (Sigma) in PBS for 15 min, and then washed twice in PBS for 10 min each time. Prior to blocking and antibody staining, cells were treated with sodium borohydride (10 mg/ml) in cytoskeleton buffer (10 mM MES, 150 mM NaCl, 5 mM EGTA, 5 mM MgCl_2_, 5 mM glucose, pH 6.1) for 15 min on ice. 3% of BSA was used to block overnight. Fixed cells were incubated with primary antibodies, wash and then followed by Alexa-conjugated secondary antibodies.

### Microscope image acquisition

Transfected cells were fully spread on fibronectin-patterned cover glass for 2–4 hours, and then loaded into a sealed live-cell imaging chamber (37°C, 5% CO_2_) for imaging in DMEM. Time-lapse TIRF images were acquired every 1 to 5 sec using an Olympus IX81 inverted TIRF microscope with a 100x (NA1.49) oil UApoN TIRF objective (Olympus), equipped with dual Photometrics Evolve512 EMCCD cameras for simultaneously acquiring GFP and RFP channels. Image acquisition was controlled by MetaMorph. Time-lapse confocal images were acquired using Perkin Elmer Spinning disk confocal with an UPlanSApo 100x oil objective (NA1.40) or UPlanSApo60x water objective (NA1.2). Adjustments to brightness and contrast were performed on FIJI.

### Image analysis

Cell membrane to cytosol intensity ratio analysis, FRAP analysis were performed using Matlab. Area of cell was defined by intensity based segmentation. Area of cell membrane was defined as 1 μm away from cell boundary in both 3D and 2D images.

### Simultaneous photoactivation and FRAP

PATagRFP tagged DAAM1 or Filamin A was co-transfected with GFP-β-actin to the cell. Single spot activation with 405 nm laser was defined using round ROI with diameters of 8 to 15 pixels. 561 nm excitation between 40 mW∙cm^-2^ to 80 mW∙cm^-2^ with various exposure was used for image acquisition. Stream acquisition rate was between 25 Hz to 33 Hz.

Imaris 7.2 spot tracking algorithm was used to track the movement of photoactivated PATagRFP molecules. A moderate searching radius of 1 μm was used.

A method has been developed to quantify the photoactivation images in Matlab in this study. A ring with R pixels from the edge of the ROI region and width ΔR was selected. The mean intensity of the ring was measured frame by frame for 1 sec. (Fig 7B, area between two yellow rings). As the DAAM1 FRAP result provided the estimation of effective diffusion coefficient of 5 μm^2^/s, the maximum distance of a DAAM1 molecule can travel within 1 second in two-dimensional plane is 20 μm. In addition to the ring area, cell boundary was applied to define the total area for total intensity integration, i.e. the total intensity of the entire cell was set as 1. The intensity versus distance from the edge of ROI at specific time post-activation was measured then plotted.

A “spreading width” was defined as the distance to the edge of the ROI at the half maximum intensity at a particular time (Fig 7C, red arrow shows the spreading width at 1 second post activation). It is a numerical estimation from the raw data instead of calculated from fitting curve, since no model was assumed. With longer intervals between each acquisition, spreading width estimation can extend further after photoactivation.

## Supporting Information

S1 Fig DAAM1 NC mutant resulted dense mesh of cytoplasmic actin. (A) Human foreskin fibroblast (HFFs) were transfected with GFP-DAAM1-WT or GFP-DAAM1-NC. The cells were fixed and stained with phalloidin and Hoechst. Regions of actin structure were enlarged. White box showed the actin structure of untransfected cells, and yellow boxes showed the transfection of respective GFP constructs. (B) Geodesic actin structure in GFP-DAAM1-NC transfected HFFs. Multiple regions of the geodesic actin were enlarged for the details. (C) Mouse embryonic fibroblasts were transfected with Lifeact-ruby and GFP-DAAM1 NC mutant (GFP-NC). The cells were treated with LatA then wash out. Aster shape of actin formed while DAAM1-NC patches were located at the center of the asters.
(TIFF)

S2 Fig Western blot showed the expression of PATagRFP-DAAM1. HeLa cells in 10 cm dishes were transfected with 2μg of respect plasmid DNAs and harvested 24 hours later for western blot detection.
(TIFF)

S3 Fig Testing and tracking of photoactivated molecules. (A) An example of simultaneous FRAP of GFP-β-actin and photoactivation of PATagRFP-DAAM1. Left image is the colored maximum intensity projection (MIP) of 1 sec post-activation. Right image is GFP-β-actin one frame before activation/bleaching. The FRAP/activation region (ROI) is highlighted in red. A kymograph of FRAP recovery is shown for the ROI region. (B) PATagRFP decays drastically over a short period of time. Measurements were taken using relatively immobile PATagRFP-Filamin A. Single spot activation with 405 nm laser was defined using round ROI with 15-pixel diameter. Acquisition using 561 nm excitation between 40 mW∙cm-2 to 80 mW∙cm-2 with various exposure times reduced the intensity to background level within 10 sec. (C) and (D) closer look of the PATagRFP-DAAM1 dynamics. A region from a non-treated MEF cell transfected with GFP-β-actin and PATagRFP-DAAM1 was enlarged. The 1 sec MIP of PATagRFP-DAAM1 was merged with the GFP-β-actin. The DAAM1 spots localized on stress fiber were highlighted using red circles. The movements of these PATagRFP-DAAM1 spots were tracked as shown in (D). (E) With longer interval between each acquisition time, PATagRFP-DAAM1 spots were found without movement. Most of these spots, highlighted in red circles, colocalized with actin nodes.
(TIFF)

S1 Movie Actin structure change during the treatment of Latrunculin A and wash off. HeLa JW cells were transfected with Ruby-Lifeact and GFP-DAAM1 24 hours before imaging. 500 nM LatA was first added to the cells. Wash off was done by replacing the LatA-containing medium to normal DMEM after 3 times washing done within 3 minutes. The movie showed the Ruby-Lifeact (magenta in merged image), GFP-DAAM1 (green in merged image) channels and the merged of the two channels. Rolling ball background subtraction was done to GFP channel to increase the contrast. The lower right region of the cell was enlarged to show the details. The first few minutes after LatA wash off was slowed down 4 times in the enlarged region. S1 Movie corresponds to Fig 2A–2E.
(AVI)

S2 Movie DAAM1-NC mutant is an active formin. H460 cells were transfected with GFP-DAAM1-NC and Ruby-Lifeact (left) or GFP-DAAM1-NCI698A and Ruby-Lifeact (right). The movie starts at 17 minutes of LatA treatment and lasts for 9 minutes. S2 Movie corresponds to Fig 3D.
(AVI)

S3 Movie Actin nodes are transient in rapamycin induced DAAM1 membrane translocation system. HeLa JW cells were transfected with Lyn11-FRB, CFP-FKBP, GFP-FKBP-DAAM1, Ruby-Lifeact and treated with 1 uM Rapamycin and 500 nM LatA. The movie starts at the beginning of LatA treatment and lasts for 40 minutes. Frames with actin nodes appearance are indicated in the movie. S3 Movie corresponds to Fig 5A.
(AVI)
