# Supplementary figures and images for: Formin DAAM1 Organizes Actin Filaments in the Cytoplasmic Nodal Actin Network
Source: PLoS One. 2016 Oct 19;11(10):e0163915. doi: 10.1371/journal.pone.0163915 (PMC5070803; doi:10.1371/journal.pone.0163915)

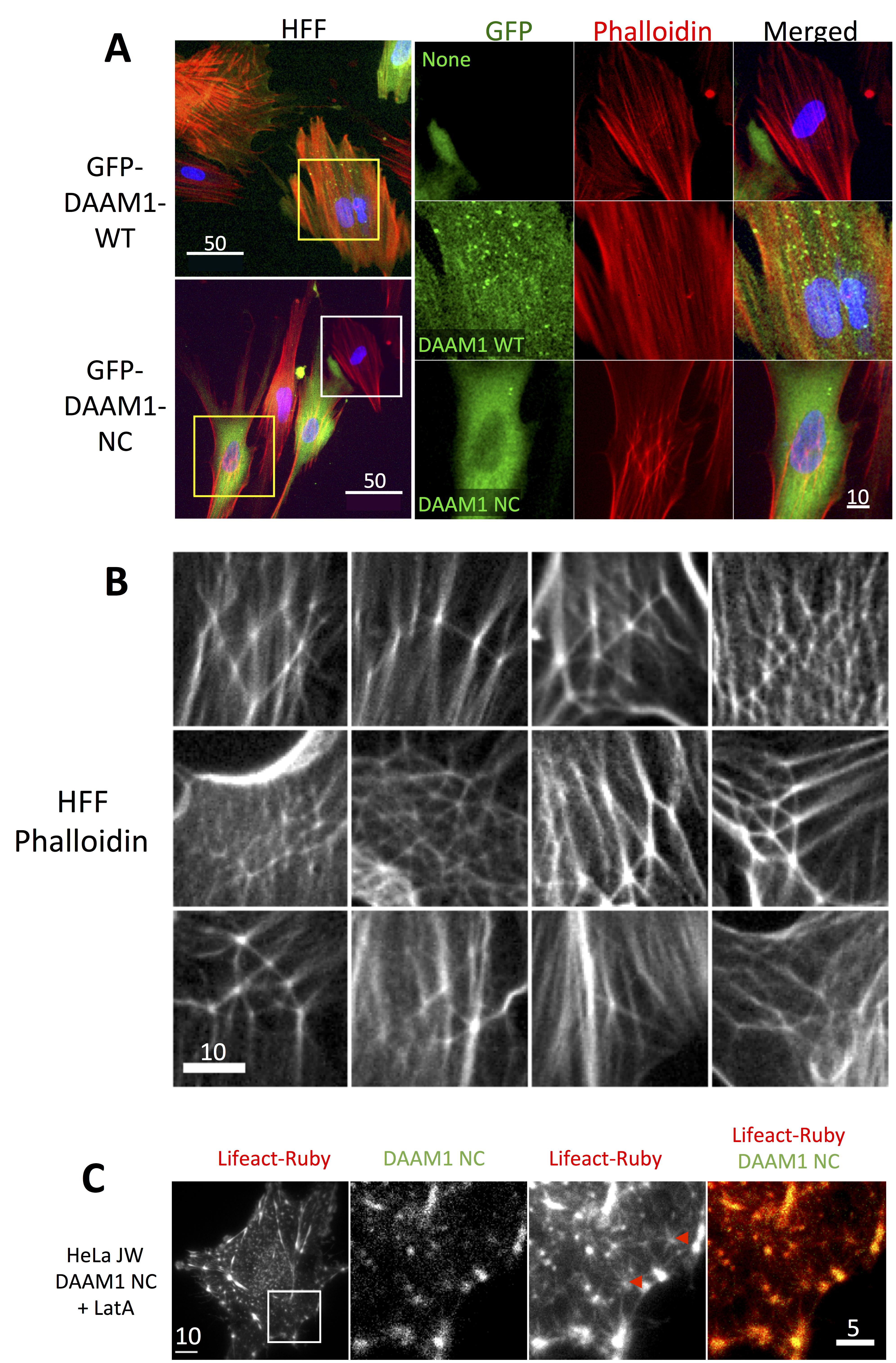

Supplement: S1 Fig — (A) Human foreskin fibroblast (HFFs) were transfected with GFP-DAAM1-WT or GFP-DAAM1-NC. The cells were fixed and stained with phalloidin and Hoechst. Regions of actin structure were enlarged. White box showed the actin structure of untransfected cells, and yellow boxes showed the transfection of respective GFP constructs. (B) Geodesic actin structure in GFP-DAAM1-NC transfected HFFs. Multiple regions of the geodesic actin were enlarged for the details. (C) Mouse embryonic fibroblasts were transfected with Lifeact-ruby and GFP-DAAM1 NC mutant (GFP-NC). The cells were treated with LatA then wash out. Aster shape of actin formed while DAAM1-NC patches were located at the center of the asters. (TIFF) [file pone.0163915.s001.tiff]

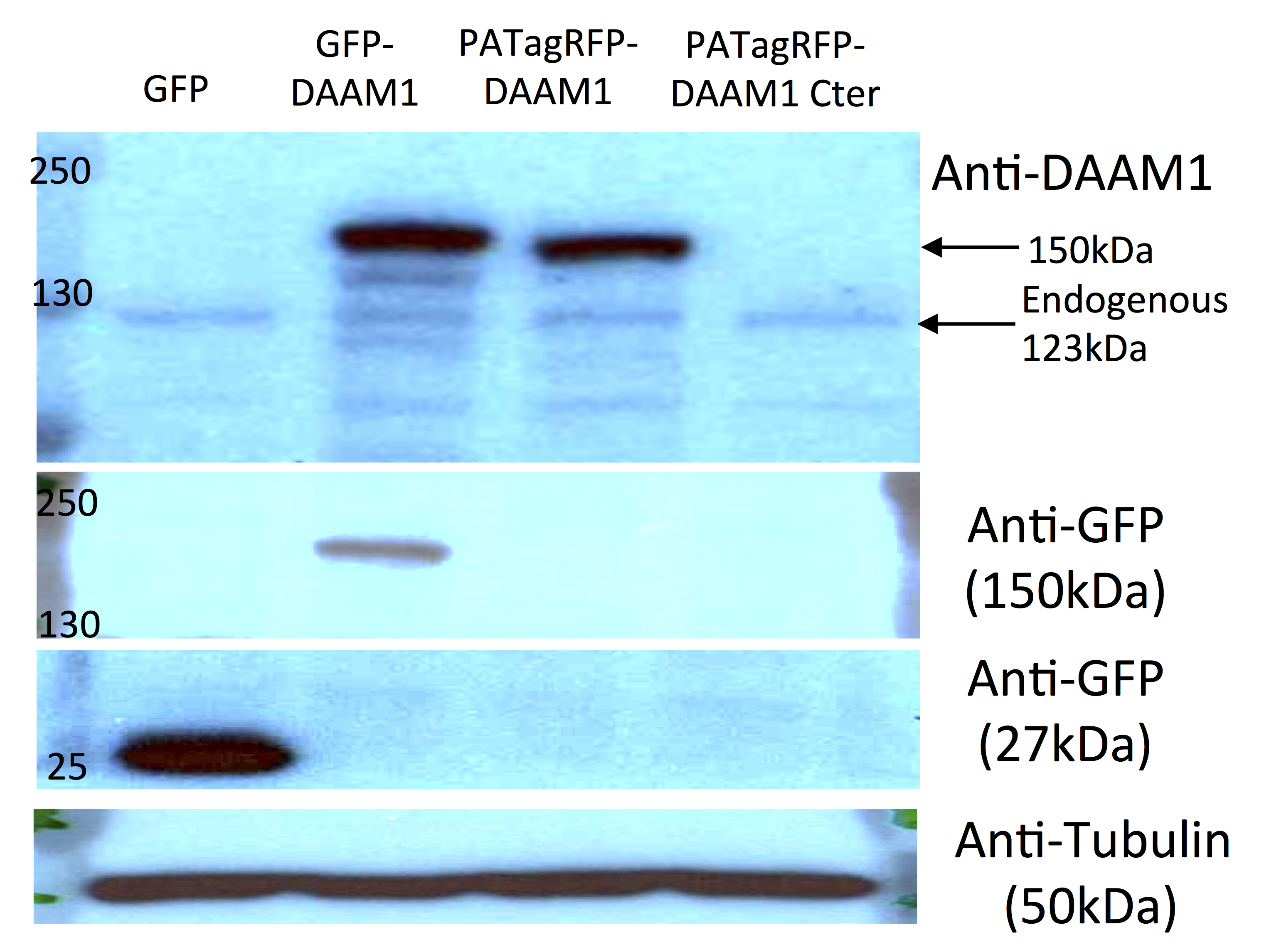

Supplement: S2 Fig — HeLa cells in 10 cm dishes were transfected with 2μg of respect plasmid DNAs and harvested 24 hours later for western blot detection. (TIFF) [file pone.0163915.s002.tiff]

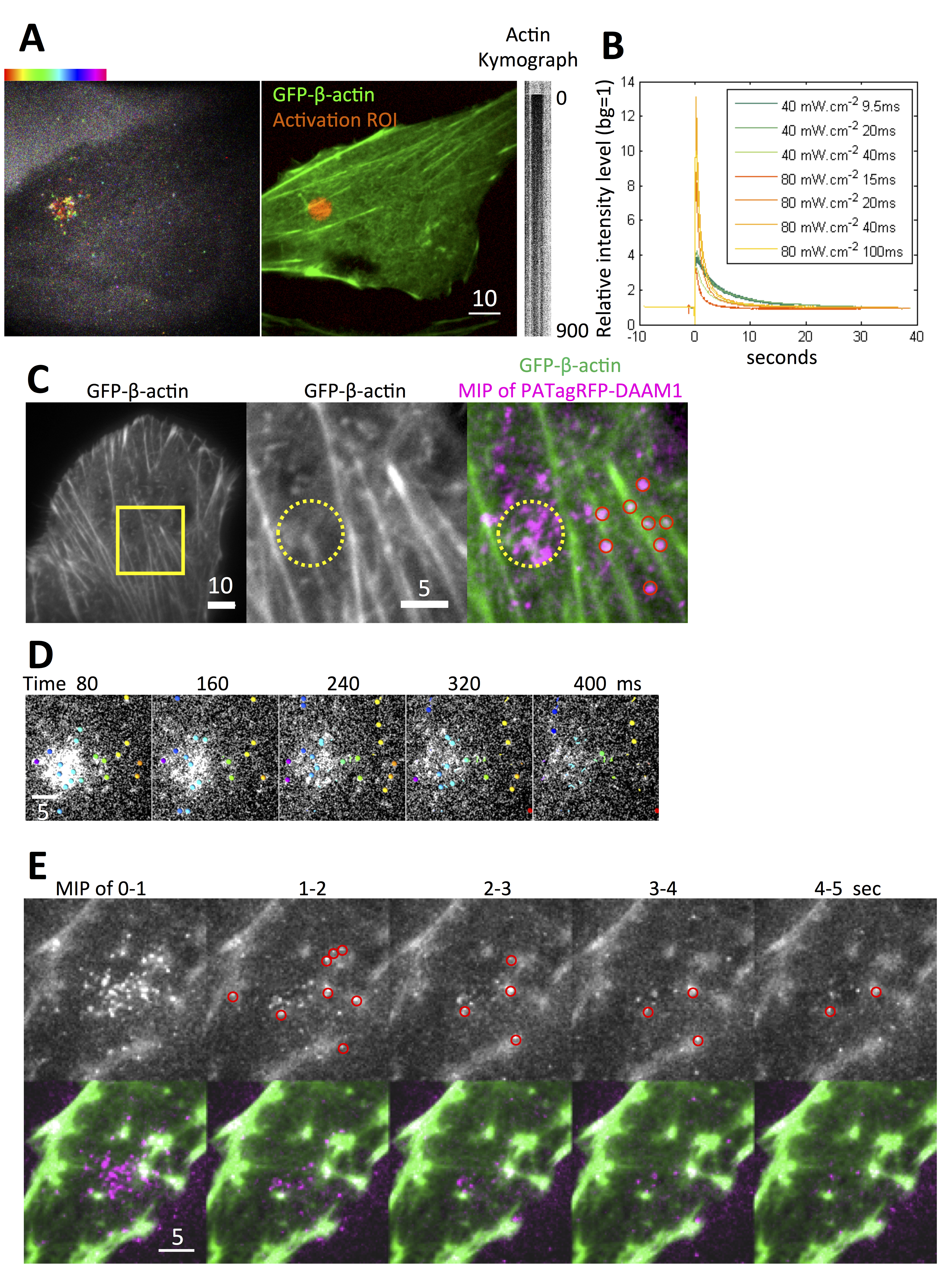

Supplement: S3 Fig — (A) An example of simultaneous FRAP of GFP-β-actin and photoactivation of PATagRFP-DAAM1. Left image is the colored maximum intensity projection (MIP) of 1 sec post-activation. Right image is GFP-β-actin one frame before activation/bleaching. The FRAP/activation region (ROI) is highlighted in red. A kymograph of FRAP recovery is shown for the ROI region. (B) PATagRFP decays drastically over a short period of time. Measurements were taken using relatively immobile PATagRFP-Filamin A. Single spot activation with 405 nm laser was defined using round ROI with 15-pixel diameter. Acquisition using 561 nm excitation between 40 mW∙cm-2 to 80 mW∙cm-2 with various exposure times reduced the intensity to background level within 10 sec. (C) and (D) closer look of the PATagRFP-DAAM1 dynamics. A region from a non-treated MEF cell transfected with GFP-β-actin and PATagRFP-DAAM1 was enlarged. The 1 sec MIP of PATagRFP-DAAM1 was merged with the GFP-β-actin. The DAAM1 spots localized on stress fiber were highlighted using red circles. The movements of these PATagRFP-DAAM1 spots were tracked as shown in (D). (E) With longer interval between each acquisition time, PATagRFP-DAAM1 spots were found without movement. Most of these spots, highlighted in red circles, colocalized with actin nodes. (TIFF) [file pone.0163915.s003.tiff]
